# Hesperetin and Naringenin promote glucagon-like peptide-1 secretion from L-cell through activation of TGR5 and alleviated type 2 diabetes

**DOI:** 10.3389/fphar.2026.1838267

**Published:** 2026-06-03

**Authors:** Chen-Yang Zhang, Zi-Lu Wang, Ze-Ju Jiang, Hanna Kim, Chang Liu, Hao Wang

**Affiliations:** 1 Beijing Key Laboratory of Diabetes Research and Care, Department of Endocrinology and Metabolism, Beijing Diabetes Institute, Beijing Tongren Hospital, Capital Medical University, Beijing, China; 2 Laboratory for Clinical Medicine, Capital Medical University, Beijing, China

**Keywords:** GLP-1 secretagogues, hesperetin, Naringenin, tgr5, type 2 diabetes

## Abstract

**Purpose:**

Glucagon-like peptide-1 (GLP-1) secretagogues offer therapeutic potential for type 2 diabetes. Bioactive compounds in citrus were supposed to be effective in treating diabetes, while exact compounds activating enteroendocrine L-cell pathways remain underexplored. This study aimed to identify citrus-derived compounds that stimulate GLP-1 secretion from L cells by screening a traditional Chinese medicine library and to elucidate the underlying cellular mechanisms.

**Methods:**

A reporter GLUTag cell line (murine L cell line), designed to introduce a luciferase into the proglucagon sequence, was utilized to screen a traditional Chinese medicine library. The effects of selected compounds on GLP-1 secretion were evaluated in both GLUTag cells and primary mouse ileac epithelial cells. Intracellular cAMP levels and calcium flux were measured to elucidate their underlying mechanism, and a dual-luciferase reporter assay was used to confirm their activation on TGR5 target. *In vivo* effect was confirmed in high-fat-diet-induced diabetic mice by oral administration.

**Results:**

Hesperetin and Naringenin significantly promoted GLP-1 secretion from L cells. Both compounds increased cAMP accumulation and intracellular calcium influx in L cells, effects that were largely absent in TGR5-deficient L cells. Subsequently, dual-luciferase reporter assays identified them as TGR5 activators. Furthermore, cellular thermal shift assay (CETSA) provided evidence that each flavonoid forms a stable complex with TGR5. Finally, in high-fat-diet-induced diabetic mice, oral administration of either compound enhanced glucose-stimulated serum GLP-1 levels and ameliorated obesity and hyperglycemia under obese diabetic conditions.

**Conclusion:**

This study demonstrated that Hesperetin and Naringenin promoted GLP-1 secretion from L-cells through TGR5 activation and alleviated type 2 diabetes.

## Graphical Abstract

**Figure:**
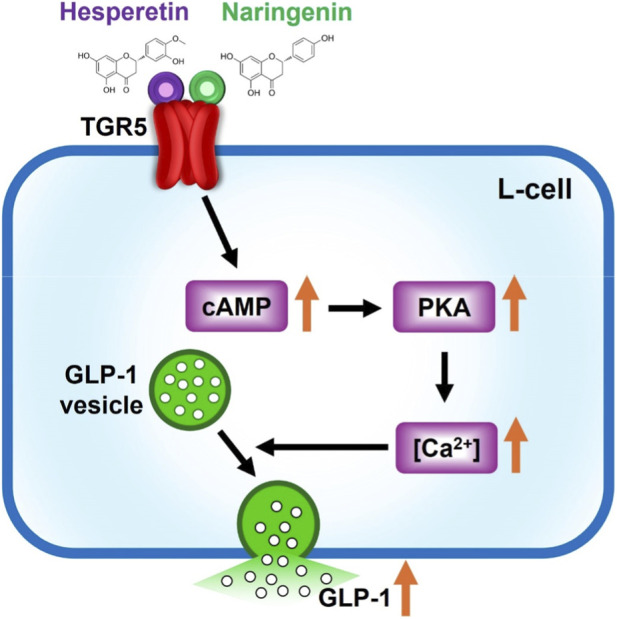
Hesperetin and Naringenin activate TGR5 in enteroendocrine L cells, elevating cAMP levels, stimulating PKA pathway, increasing calcium influx, and enhancing GLP-1 secretion, thereby improving obesity and glucose metabolism under obese diabetic conditions.

## 1 Introduction

Type 2 diabetes (T2D) remains a global health crisis (An et al., 2025). Targeting the secretion of anorectic and antidiabetic gut peptides has become a major focus in drug development, as growing evidence suggests that enhancing the release of glucagon-like peptide (GLP)-1 from intestinal L cells may confer therapeutic benefits in individuals with diabetes and obesity (Mlynarska et al., 2025; Nauck et al., 2021). GLP-1 potentiates glucose-dependent insulin release and satiety, are associated not only with improved blood glucose control and reduced incidence of hypoglycemia but also with significant weight reduction (Andersen et al., 2018). Identifying and validating pharmaceutical strategies to enhance GLP-1 secretion are central to many ongoing L cell-targeting research programs. The G protein-coupled receptor TGR5 (also called GPBAR1) is a key regulator of GLP-1 release, activating cAMP/PKA signaling and enhanced voltage-gated Ca^2+^ influx in L-cells (Sorrentino et al., 2020). Although bile acids are canonical TGR5 agonists, their clinical utility is constrained by hepatotoxicity (Jin et al., 2024). While synthetic GLP-1 analogs are clinically established, their limitations including cost, side effects, and suboptimal oral bioavailability, motivate the search for natural compounds that safely amplify L-cell secretion (Zhang et al., 2025).

Traditional Chinese Medicine has been increasingly recognized for its potential to manage obesity and diabetes (Chen et al., 2023). Many bioactive compounds in citrus have been reported to be effective in metabolic syndrome such as diabetes and obesity (Visvanathan and Williamson, 2023; Sharma et al., 2015). Mediterranean diet intervention with Citrus Bioflavonoids reduced inflammation in people with T2D (Al-Aubaidy et al., 2021). Flavanones in Citrus such as Hesperetin and Naringenin exhibited anti-oxidant, anti-inflammation and antidiabetic properties, but their direct effects on GLP-1 secretion were uncharacterized (Saini et al., 2022; Gandhi et al., 2020). Our research combined high-throughput screening in reporter cells, mechanistic studies in L-cell models, and *in vivo* validation in diabetic mice to identify Hesperetin/Naringenin as potent GLP-1 secretagogues and elucidate their activation of TGR5-dependent cAMP/PKA and Ca^2+^ pathways.

## 2 Materials and methods

### 2.1 Materials

The Traditional Chinese Medicine Library (L8300) consisting of 1,561 compounds was purchased from Selleckchem (Shanghai, China). Hesperetin and Naringenin used *in vivo* and *in vitro* studies was purchased from MACKLIN (Shanghai, China). H89 (HY-15979), Nifedipine (HY-B0284), GW 4064 (HY-50108), INT777 (HY-15677), TAK-875 (HY-10480), GSK1292263 (HY-12066), TUG-891 (HY-100881) and Exendin-(9-39) amide (HY-P0264) were purchased from MedChemExpress (Shanghai, China). Aprotinin and Diprotin A (DPP4 inhibitor) were purchased from MACKLIN (Shanghai, China). Protease inhibitor cocktail tablets were purchased from Roche (Mannheim, Germany). Anti-TGR5 antibody (ab72608) was purchased from Abcam (Cambridge, United Kingdom). Mouse GLP-1 ELISA kit was purchased from Crystal Chem (81506, Elk Grove Village, United States). Ultrasensitive Mouse Insulin ELISA kit was purchased from Mercodia (10-1249-01, Uppsala, Sweden). cAMP ELISA Kit was purchased from Elabscience (E-EL-0056, Wuhan, China). CCK-8 kit was purchased from TransGen Biotech (Beijing, China).

### 2.2 Animals and treatment

The animal experiments were approved by the Ethical Review Committee of the Institute of Zoology, Capital Medical University, China (TRLAWEC2025-08). Six-week-old male C57BL/6J mice were purchased from GemPharmatech Co. Ltd (Nanjing, China). The mice were kept in a temperature-controlled room at 22 °C ± 2 °C on a 12-h light/dark cycle and were fed with a normal diet (ND) or high-fat diet (HFD) (comprising 60% fat, 20% carbohydrate, and 20% protein by caloric content; Research DIETS, D12492, New Brunswick, NJ, United States) for 16 weeks. The mice fed with ND were used as control group in the experiment. Age- and bodyweight-matched 16-week HFD-induced type 2 diabetic mice (Surwit et al., 1995; Cabello-Olmo et al., 2023; Phillips et al., 2022) used in the experiment were randomly divided into three groups: Group 1 (Vehicle, n = 6), Group 2 (Hesperetin at 100 mg/kg bodyweight, n = 6) and Group 3 (Naringenin at 100 mg/kg bodyweight, n = 6). The dosage was selected based on previous studies demonstrating the efficacy and safety of these flavonoids at this concentration in rodent models of metabolic disease (Yu et al., 2024; Wang et al., 2020). For the short-term administration study, vehicle, Hesperetin or Naringenin was administered orally 40 min before oral glucose tolerance test (OGTT). Exendin-(9-39) (Ex9) was dissolved in sterile saline and administered via intraperitoneal injection at a dose of 50 μg/mouse, 30 min before the oral glucose challenge (Burmeister et al., 2017; Chambers et al., 2017). For the long-term administration study, vehicle, Naringenin or Hesperetin was administered orally once daily over a 4-week period, age-matched ND fed mice administered with vehicle were used as normal control. Body weight was measured at 1-week intervals. Food intake was measured specifically during the final week of the study and insulin tolerance test (ITT) was detected at the last day. Oral glucose tolerance test (OGTT) was performed 3 days after the ITT. All mice were fasted for 12 h before experiment of OGTT, ITT and OGTT were detected as described previously (Yuan et al., 2024). Blood samples were collected from the angular vein mixed with an inhibitor (including Aprotinin, Diprotin A and protease inhibitor cocktail tablets) before and 10, 60 min after oral-administration of 2 g/kg D-glucose. Serum glucose, insulin and GLP-1 were measured as described previously (Yuan et al., 2024).

### 2.3 Cell culture

The mouse enteroendocrine cell line, GLUTag (Drucker et al., 1994) (a gift kindly provided by Dr. Daniel Drucker) and HEK293 cells (Wang et al., 2024) were all maintained in DMEM (4.5 g/L D-Glucose) (Gibco, United States), supplemented with 10% fetal bovine serum (Gibco, United States), 100 IU penicillin and 100 μg/mL (Beyotime, China) in a 95% air and 5% CO_2_ atmosphere at 37 °C. An equal number of GLUTag cells were plated in 24-well plates and allowed to adhere until the cells are about 80% confluent for GLP-1 secretion assay.

### 2.4 Construction of GLP-1 reporter cell line

Based on Sorina Andreea Anghel’s recent findings (Anghel et al., 2022), we utilized the pLVX-Puro lentiviral expression vector (XIEBHC Biotechnology, Beijing, China) to generate the pLVX-mGLP-1-NanoLuc-Puro plasmid. The mGLP-1-NanoLuc sequence was synthesized via full gene synthesis as described elsewhere (Anghel et al., 2022). Using XbaI and NotI restriction enzymes, the pLVX-Puro backbone was linearized. The primers designed for this process were: XbaI_forward: CTC​GAG​ACT​AGT​TCT​AGA​gcc​acc​ATG​AAG​ACC​ATC​TAC​TTC​GTG​GCC; NotI_reverse: GGG​AGA​GGG​GCG​GGA​TCC​GCG​GCC​GCT​CAC​TTC​TTG​TCG​GTG​ATC​TTG. The mGLP-1-NanoLuc insert was then ligated into the pLVX-Puro vector. The mGLP-1-NanoLuc insert was then ligated into the linearized vector via the corresponding restriction sites. Successful cloning was confirmed by restriction digestion and Sanger sequencing. For stable expression, a second-generation lentiviral packaging system was employed. HEK293T cells were co-transfected with the transfer plasmid pLVX-mGLP-1-NanoLuc-Puro, along with the packaging plasmid psPAX2 and the envelope plasmid pMD2. G (both from XIEBHC Biotechnology, Beijing, China). The culture medium was replaced with fresh medium 6 h after transfection. After replacing the medium 6 h post-transfection, viral supernatant was harvested at 48 h, followed by purification and filtration through a 0.22 μm PES membrane (Merk, Darmstadt, Germany). GLUTag cells at 40% confluence were transduced with the lentiviral particles in the presence of LV-Enhance for 24 h. Thereafter, the medium was replaced with fresh medium, and the cells were cultured for an additional 48 h. Stable polyclonal populations were selected using three rounds of puromycin treatment (2 μg/mL) and maintained in medium containing the same concentration of puromycin.

### 2.5 Drug screening

The L8300-Traditional Chinese Medicine Library containing 1,561 compounds pre-dissolved in DMSO (10 mM) was used to screen for GLP-1 secretagogues at 1 μM final concentration. GLP-1 reporter cells were seeded in Poly-L-lysine (Sigma) coated 96-well plate and were cultured for 48 h until the cells were about 90% confluent. For secretion assay, cells were washed and incubated 10 min in Krebs-Ringer bicarbonate buffer (KRBB) prior to be treated with KRBB supplemented with 0.1% BSA, 10 mM Glucose, and DMSO or 10 μM of each compound for 1.5 h. The secreted medium was transferred into a white 96 well plates containing coelenterazine 5 μM (NanoLuc substrate) to detect the fluorescence of supernatant. The compounds in the library were divided into 10 per group to detect the fluorescence of supernatant. Then, the groups which promoted secretion of GLP-1 greater than 1.2-fold were picked out and screened one by one. Finally, the top-ranked drugs with promotion of GLP-1 greater than 1.5-fold and no cytotoxicity were selected as candidate drugs.

### 2.6 Cell viability assay

The reporter cells or GLUTag cells were seeded into a 96-well plate and allowed to adhere until the cells are about 80% confluent. Then reporter cells were treated with 1 μM of different compounds at the same time or the equivalent amount of solvent (DMSO) for 1.5 h. While GLUTag cells were incubated with 0.01 μM, 0.1 μM, 1 μM, 10 μM, 100 μM of Hesperitin and Naringenin at the same time or the equivalent amount of solvent (DMSO) for 1.5 h. After treatments, CCK-8 was added to the culture medium, and then the cells were incubated for 2 h at 37 °C in an incubator. The absorbance at 450 nm was measured using an automatic plate reader.

### 2.7 Primary intestinal epithelial cell culture and treatment

The primary intestinal epithelial cell (PIECs) was isolated from murine ileum as described previously (Yuan et al., 2024). The intestines of mice were sacrificed by cervical dislocation and placed into ice-cold DPBS. The ileum was opened and washed in DPBS and then sliced into 1–2 mm^2^ pieces. The tissue pieces were treated in 4 mM EDTA buffer for 30 min on ice. Tissue pieces were centrifuged and forcefully resuspended in cold 5% FBS in DPBS. The resuspension/sedimentation process was repeated 5-6 times, then they were centrifuged at 200 × g and resuspended in DMEM (25 mM glucose) supplemented with 10% FBS, 2 mM glutamine, 1x Penicillin-Streptomycin solution. 24-well plates covered with 1% v/v Matrigel were used to plate intestinal cell/crypt suspensions.

### 2.8 GLP-1 secretion assay

After washed and incubated 30 min in Krebs-Ringer bicarbonate buffer (KRBB) without glucose, GLUTag cells or PIECs were then treated with KRBB supplemented with 0.1% BSA, 20 mM Glucose and 100 μM Diprotin A with or without chemical compounds for 1.5 h as described previously (Yuan et al., 2024). Then the medium and cell lysate were collected to detect GLP-1 concentration by using GLP-1 ELISA kit.

### 2.9 cAMP measurement

cAMP measurement was performed as described previously (Jiang et al., 2025). To measure cAMP levels in GLUTag cells, 24-well plate with 80% cell confluence was used. After washed and incubated 30 min in PBS, cells were then treated with KRBB supplemented with 0.1% BSA, 20 mM Glucose and DMSO or Hesperetin/Naringenin 10 μM for 1.5 h. Subsequently, they were lysed immediately, and the cellular cAMP level was detected with a cAMP ELISA kit.

### 2.10 Live‐cell Ca^2+^ imaging

GLUTag cells were seeded onto poly‐L‐lysine‐coated glass base dishes and cultured for 24 h. For calcium imaging, cells were loaded with 2 μM Fluo‐4 AM (Dojindo, Tokyo, Japan) in standard KRBB at 37 °C for 30 min, followed by washing with dye‐free KRBB. After a subsequent 10-min equilibration in the same buffer, the cells were stimulated with KRBB containing 10 mM glucose. High‐resolution live‐cell imaging was performed using a DeltaVision Ultra microscope system (GE Healthcare, United States) (Jiang et al., 2025). The Fluo‐4 AM probe was optically excited at a 488 nm wavelength, with emitted fluorescence signals detected at 525 nm. Time-lapse acquisition started 60 s before stimulation, with images captured every 30 s. Image sequences were analyzed in ImageJ, and calcium dynamics were expressed as the normalized fluorescence ratio (F/F_0_), where F is the instantaneous fluorescence intensity and F_0_ represents the mean baseline fluorescence from the initial 30-s recording.

### 2.11 Plasmid construction and transfection

For expression of human FXR, human TGR5, human GPR119, human GPR40 and human GPR120, the ORF region of them was inserted into pcDNA3.1 vector (Invitrogen, CA, United States) (Wang et al., 2023). The pGL vector encoding the Firefly luciferase gene (Promega, Madison, WI, United States) was used for generation of luciferase reporter plasmids. The FXR response element was cloned into the pGL4.23 vector (pFXRE-luc), the CRE was cloned into the pGL4.29 vector (pCRE-luc), and the NFAT was cloned into the pGL4.30 vector (pNFAT-luc). For GPCR activity assay, 293 cells were transfected with the plasmid using Lipofectamine 3,000 (Thermo Fisher Scitific, MA, United States) transfection reagent and they were analyzed 48 h later. For TGR5 knockdown experiment, GLUTag cells were transfected with 100 nM non-targeting siRNA or siRNA targeting mouse TGR5 (GCC​TAC​TAC​TAG​CCG​GGC​T) using Lipofectamine RNAiMAX (Thermo Fisher Scitific, MA, United States) following the manufacturer’s instructions for 2 days.

### 2.12 Dual-luciferase reporter assay

The Dual-Luciferase Reporter Assay was conducted with the Firefly Luciferase Reporter® assay system (Promega Corporation, Madison, WI, United States). For human TGR5 and GPR119 luciferase assay, 293T cells were co-transfected with pCRE-luc, pTGR5 or pGPR119, and pRL-TK; for human GPR40 and GPR120 luciferase assay, 293T cells were co-transfected with pNFAT-luc, GPR40 or GPR120, and pRL-TK; for human FXR luciferase assay, 293T cells were co-transfected with pFXRE-luc, pFXR, and pRL-TK. The pRL-TK plasmid encoding the Renilla luciferase gene (Promega, Madison, WI, United States) was used as an internal control to normalize the firefly luciferase signal across all samples. DMSO, Hesperetin, Naringenin or related GPCR agonist were added 24 h later. Cells were harvested after 16 h and subjected to luciferase analysis using the Dual Luciferase Reporter Assay Kit (DL101-01, Vazyme, Nanjing, China) on Infinite M Plex system (Tecan, Männedorf, Switzerland). All reporter assays were repeated at least three times.

### 2.13 Real-Time quantitative PCR

RNA was extracted and reverse transcribed as described previously (Wang et al., 2023). Real-Time quantitative PCR was performed on a LightCycler 480 Real-Time PCR System (Roche, Basel, Switzerland) using SYBR Green I Master Mix reagent (Roche) with the primers. The target mRNA expression level was calculated and normalized to Rplp0/36B4 mRNA level. The primers for mouse genes were as follows: TGR5 forward primer 5′- CCT​GGC​AAG​CCT​CAT​CGT​C-3′ and reverse primer 5′- AGC​AGC​CCG​GCT​AGT​AGT​AG-3′, Rplp0/36B4 forward primer 5′-GGC​CCT​GCA​CTC​TCG​CTT​TC-3′ and reverse primer 5′-TGC​CAG​GAC​GCG​CTT​GT-3′.

### 2.14 Western blot

The cells were collected and lysed using a lysis buffer containing 150 mmol/L NaCl, 20 mmol/L Tris-HCl (pH 7.5), 10 mmol/L EGTA, 1 mmol/L MgCl_2_, 1% Triton X-100, 1 mmol/L phenylmethylsulfonyl fluoride (PMSF), and complete protease inhibitor cocktail. Immunoblotting analyses were conducted as previously described (Wang et al., 2023). Finally, the protein bands were detected using the enhanced chemiluminescence (Amersham Biosciences, United States) and the LAS-500 chemiluminescence detection system (GE Healthcare Bioscience, United States). Quantification of protein levels were analyzed using ImageJ software.

### 2.15 Cellular thermal shift assay (CETSA)

293 cells transfected with human TGR5 were treated with 10 μM Hesperetin, or 10 μM Naringenin or vehicle for 2 h, then they were resuspended in PBS and equally divided into 7 parts. Each part was heated for 3.5 min under different temperatures (41, 44, 47, 50, 53, 56, and 59 °C), and cooled at −80 °C overnight. The samples were then allowed to equilibrate at room temperature for 5 min before being subjected to liquid nitrogen cooling for an additional 5 min, a procedure that was repeated 3 times. After that, the lysates were centrifuged at 14,000 rpm, and the TGR5 protein levels were detected through Western blot analysis.

### 2.16 Statistical analysis

Statistical analysis was performed by GraphPad Prism 9. For comparison between the two groups, a Mann-Whitney U-test was used. For multiple comparisons, one-way ANOVA with Turkey’s test was used. Statistical significance was set at *p < 0.05, **p < 0.01, and ***p < 0.001.

## 3 Results

### 3.1 Hesperetin and Naringenin were identified as novel GLP-1 secretagogues by using a reporter cell line for GLP-1 secretion

**FIGURE 1:**
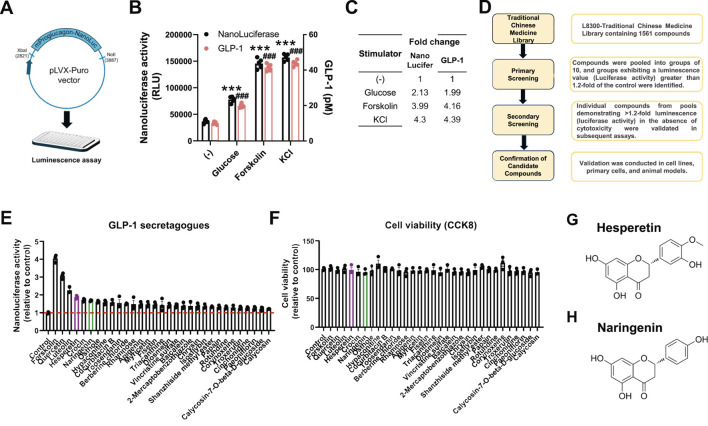
Screening GLP-1 secretagogues from traditional Chinese medicine library by using a GLP-1-NanoLuciferase reporter GLUTag cell line. **(A)** Schematic of construction of GLP-1-NanoLuciferase reporter GLUTag cell line. **(B)** NanoLuciferase GLUTag cells were incubated with assay buffer containing DMSO (control), 20 mΜ Glucose, 10 μΜ forskolin and 50 mM KCl. Luciferase activity (luminescence) was immediately detected, secreted physiological GLP-1 were measured with GLP-1 ELISA kit (n = 5). ***p < 0.001 or ^###^ *p* < 0.001 vs. the control group by one-way ANOVA with Turkey’s test. **(C)** Similar fold changes of secreted NanoLuciferase activity and corresponding physiological GLP-1 concentrations in response to different secretagogues. **(D)** Strategy flowchart for screening compounds that stimulate GLP-1 secretion by using reporter cell line. **(E,F)** NanoLuciferase activity assay for GLUTag cells treated with assay buffer plus DMSO (control) or 10 μM different compounds, compounds that exhibited a greater than 1.2-fold increase with statistic significant were shown, the red dotted line represented the values of the control group **(E)**, while cell viability was detected by CCK-8 assay **(F)** (n = 3). *p < 0.05 by Mann-Whitney U-test. **(G-H)** Chemical structure of hesperetin (G) and naringenin **(H)**. Data are expressed as the mean ± SEM.

NanoLuciferase GLUTag cell line was constructed following procedure showed in Method (Figure 1A). Glucose, forskolin and KCl was independently incubated with these cells to stimulate GLP-1 secretion for validating the accurate of the reporter cell line, the result showed that NanoLuciferase activity in culture media was in accordance with physiological GLP-1 concentration in culture media measured by ELISA kits, which confirmed the reliability of the cell line (Figures 1B,C). Then we screened candidate of GLP-1 secretagogues from L8300-Traditional Chinese Medicine Library containing 1,561 compounds using methods listed in Figure 1D. Briefly, we initially divided all compounds into several groups of 10 compounds for primary screening. Then, groups demonstrating GLP-1 secretion exceeding 1.2-fold relative to the control were selected for subsequent analysis. Compounds within these positive groups were then subjected to individual validation assays, accompanied by cytotoxicity assessment. Finally, we found 30 compounds in the library that increase NanoLuciferase more than 1.2-fold compared with the control without cytotoxicity (Figures 1E,F). Among these selected compounds, we chose the top 5 compounds as the candidates including Quercetin (3.07-fold), Luteolin (2.25-fold), Hesperetin (1.8-fold), Crocin (1.71-fold) and Naringenin (1.68-fold). Since Quercetin, Luteolin and Crocin have been reported to increase GLP-1 secretion (Anghel et al., 2022; Kwon and Choi, 2018; Zhao et al., 2023), therefore, we chose Hesperetin (pink color) and Naringenin (green color) as the better candidates for subsequent study. By coincidence, Hesperetin and Naringenin are both belong to the flavonoid compounds, which has the similar chemical structures as shown in Figures 1G,H respectively. These results demonstrated flavonoid compounds Hesperetin and Naringenin were identified as GLP-1 secretagogues by using a reporter NanoLuciferase GLUTag cell line.

### 3.2 Hesperetin and Naringenin promoted glucose-stimulated GLP-1 secretion from L-cells

**FIGURE 2:**
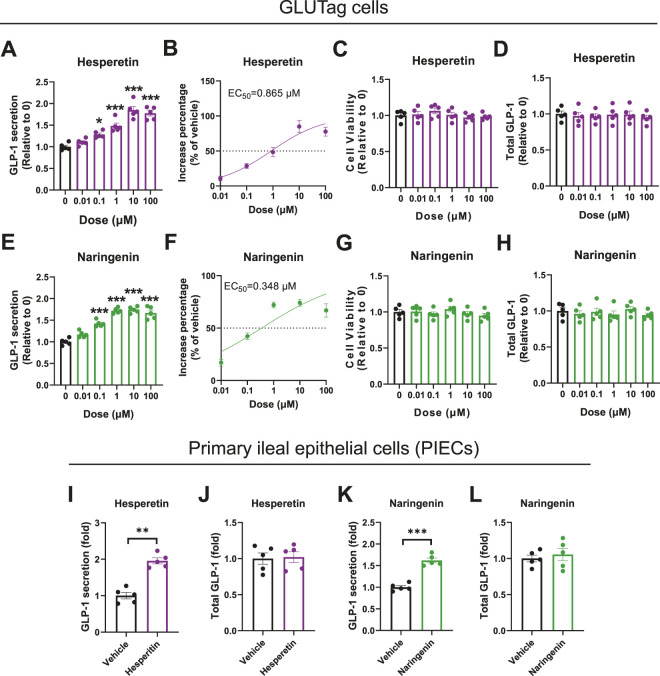
Hesperetin and Naringenin amplifies glucose-stimulated GLP-1 secretion. **(A–D)** GLP-1 secretion **(A)**, EC_50_ was 0.865 μM for Hesperetin **(B)**, Cell viability **(C)** and Total GLP-1 **(D)** from GLUTag cells in response to different concentrations of Hesperetin (n = 6). *p < 0.05, ***p < 0.001 vs. the control group by one-way ANOVA with Turkey’s test. **(E–H)** GLP-1 secretion **(E)**, EC_50_ was 0.348 μM for Hesperetin **(F)**, Cell viability **(G)** and Total GLP-1 **(H)** from GLUTag cells in response to different concentrations of Naringenin (n = 6). ***p < 0.001 vs. the control group by one-way ANOVA with Turkey’s test. **(I,J)** GLP-1 secretion and total GLP-1 from primary intestinal epithelial cells in response to 10 μM Hesperetin. **(K,L)** GLP-1 secretion and total GLP-1 from primary intestinal epithelial cells in response 10 μM Naringenin. **p < 0.01, ***p < 0.001 by Mann-Whitney U-test, ns means not significant. Data are expressed as the mean ± SEM.

To confirm roles of Hesperetin and Naringenin on physiological GLP-1 secretion, we used GLUTag cell line and primary intestinal epithelial cells of mice to determine the effect of Hesperetin and Naringenin on GLP-1 secretion. We first treated GLUTag cells with glucose plus Hesperetin (0.01, 0.1, 1, 10 and 100 µM) or Naringenin (0.01, 0.1, 1, 10 and 100 µM). We show that Hesperetin (0.1–100 μM) significantly stimulated GLP-1 secretion from GLUTag cells with an EC50 of 0.865 μM and a maximal effect at 10 μM (Figures 2A,B), without affecting cell viability or total GLP-1 content (Figures 2C,D). In addition, Naringenin at concentrations ranging from 0.1 to 100 μM stimulated GLP-1 secretion from GLUTag cells with an EC_50_ of 0.348 μM and a maximal effect at 10 μM (Figures 2E,F), without affecting cell viability or total GLP-1 content (Figures 2G,H). Based on these results, we chose 10 μM Hesperetin and 10 μM Naringenin for subsequent cellular experiments. We next examined the stimulatory effects of Hesperetin and Naringenin on GLP-1 secretion in mouse primary ileal epithelial cells (PIEC). Similar as GLUTag cells, Hesperetin and Naringenin significantly enhanced GLP-1 secretion from PIEC with no effect on total intracellular GLP-1 content, suggesting that their effect on L-cells was physiologically relevant (Figures 2I–L). These results indicated that Hesperetin and Naringenin potently stimulated GLP-1 secretion from L-cells.

### 3.3 Hesperetin and Naringenin induced GLP-1 secretion through activation of cAMP-PKA pathway and increased intracellular calcium concentration in murine L cells

**FIGURE 3:**
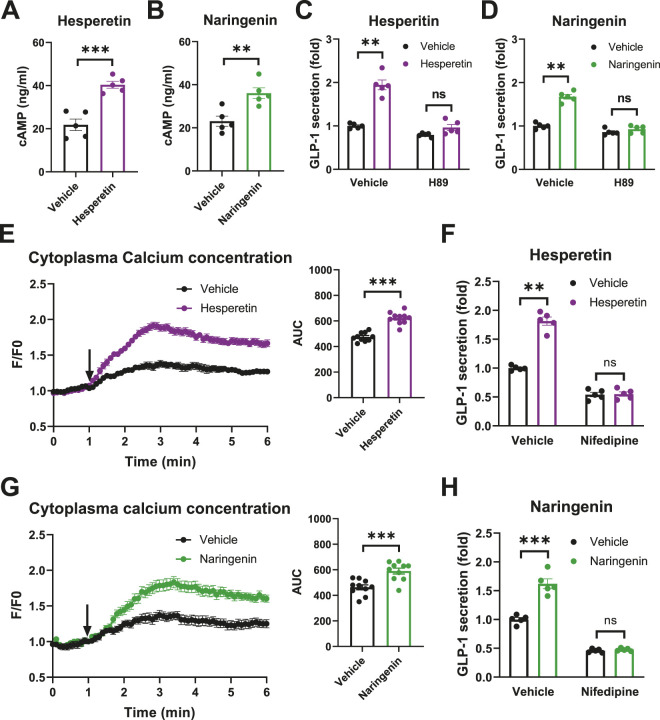
Hesperetin and Naringenin increased cAMP level and [Ca^2+^]_i_ after glucose stimulation in murine L cells. **(A,B)** Intracellular cAMP levels from GLUTag cells in response to 10 μM Hesperetin **(A)** or 10 μM Naringenin **(B)** (n = 5). **(C,D)** GLP-1 secretion from GLUTag cells in response to 10 μM Hesperetin **(C)** or 10 μM Naringenin **(D)** with 10 μM H89 or vehicle (n = 5). **(E)** The intracellular calcium concentration ([Ca^2+^]_i_) of GLUTag cells stimulated with 20 mM glucose plus vehicle or 10 μM Hesperetin was detected by using a Ca^2+^ indicator, Fluo‐4 (n = 10). The change of fluorescence after stimulation was normalized to the basal fluorescence (ΔF/F_0_) was measured by area under the curve (AUC). **(F)** GLP-1 secretion from GLUTag cells in response to 10 μM Hesperetin with 5 μM Nifedipine or vehicle (n = 5). **(G)** The intracellular calcium concentration ([Ca^2+^]_i_) of GLUTag cells stimulated with 20 mM glucose plus vehicle or 10 μM Naringenin was detected by using a Ca^2+^ indicator, Fluo‐4 (n = 10). The change of fluorescence after stimulation was normalized to the basal fluorescence (ΔF/F_0_). **(H)** GLP-1 secretion from GLUTag cells in response to 10 μM Naringenin with 5 μM Nifedipine or vehicle (n = 5). Black arrows indicated the initial time of glucose stimulation. **p < 0.01, ***p < 0.001 by Mann-Whitney U-test, ns means not significant. Data are expressed as the mean ± SEM.

The secretion of GLP-1 is closely related to cAMP/PKA signaling pathway and intracellular calcium concentration (Kuhr et al., 2021). We therefore examined the effect of Hesperetin and Naringenin on cAMP in L cells firstly. We found that incubation of GLUTag cells with Hesperetin and Naringenin markedly increased intracellular cAMP accumulation (Figures 3A,B). Since PKA is directly activated by cAMP, we next examined whether the cAMP/PKA pathway mediated Hesperetin and Naringenin induced GLP-1 secretion. We found that inhibiting the activity of PKA by H89 (a PKA inhibitor) abrogated Hesperetin and Naringenin-induced GLP-1 secretion (Figures 3C,D). Intracellular calcium concentration ([Ca^2+^]_i_) also plays significant roles in GLP-1 secretion (Kuhr et al., 2021). We examined the dynamics of calcium influx induced by short‐term treatment of Hesperetin and Naringenin in GLUTag cells. We found both Hesperetin and Naringenin obviously elevated glucose-induced [Ca^2+^]_i_, with a significant enhancement of the area under curve (AUC) (Figures 3E,G). However, after incubated with Nifedipine, a Ca^2+^ channel inhibitor, the stimulating effect of Hesperetin and Naringenin on GLP-1 secretion were both abolished (Figures 3F,H). These results indicated that Hesperetin and Naringenin promoted GLP-1 secretion through activation of cAMP/PKA/[Ca^2+^]_i_ signaling pathway in L cells.

### 3.4 Hesperetin and Naringenin triggered activation of TGR5 receptor

**FIGURE 4:**
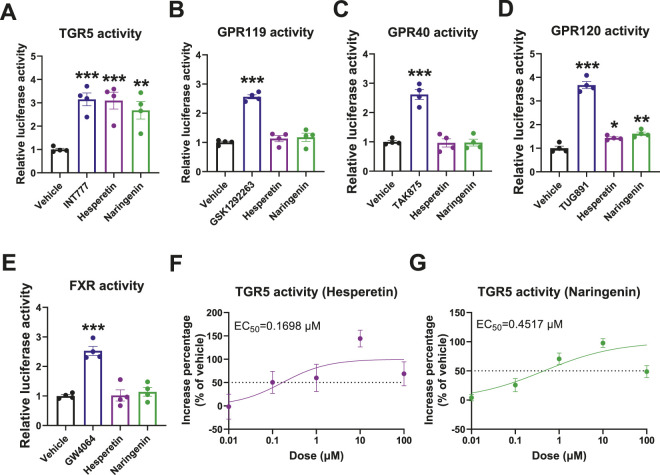
**(A-E)** Dual-luciferase reporter assay of the activation of different GPCRs containing TGR5 **(A)**, GPR119 **(B)**, GPR40 **(C)**, GPR120 **(D)** and bile acid receptor FXR **(E)** was conducted as described in method. 293T cells transfected with the reporter plasmid, the expression plasmids and the internal control plasmid were treated with vehicle, 10 μM Hesperetin, 10 μM Naringenin or related agonists such as 30 μM INT-777 for TGR5, 1 μM GSK1292263 for GPR119, 10 μM GW 4064 for FXR, 0.1 μM TAK-875 for GPR40 and 10 μM TUG891 for GPR120 respectively (n = 4). **(F-G)** The hTGR5 targeted CRE-luciferase activity of Hesperetin **(F)** and Naringenin (G) at the concentration range of 0.01-100 μM, EC50 was 0.1698 μM and 0.4517 μM for Hesperetin and Naringenin respectively (n = 3). *p<0.05, **p<0.01, ***p<0.001 vs vehicle by one-way ANOVA with Turkey’s test. Data are expressed as the mean ± SEM.

Since Hesperetin and Naringenin activated the cAMP/PKA/[Ca^2+^]_i_ signaling pathway and promoted GLP-1 secretion in L cells, this activation effect closely resembled those resulting from G protein coupled receptor (GPCR) activation, so we speculated them to be GPCR agonists. To further elucidate the target of Hesperetin and Naringenin, dual-luciferase reporter assay was conducted. We found that both Hesperetin and Naringenin significantly increased TGR5 activity, while Hesperetin appeared comparable to INT777 (a TGR5 agonist), was higher than that of Naringenin (Figure 4A). By contrast, neither of them enhanced the relative luciferase activity of GPR40 and GPR119 (Figures 4B,C). Although Hesperetin and Naringenin slightly enhanced GPR120 activity, its effect was far less potent compared to that on TGR5 (Figure 4D). Since both TGR5 and FXR are key bile acid-activated receptors involved in regulating GLP-1 secretion, we also examined the effect of Hesperetin and Naringenin on activation of FXR and found that they did not change it (Figure 4E). Subsequent analysis confirmed that Hesperetin and Naringenin dose dependently stimulate luciferase reaction through TGR5 mediated pathway, the TGR5 EC50 is 0.1698 μM for Hesperetin (Figure 4F) and 0.4517 μM for Naringenin (Figure 4G) respectively, which is similar as those in GLUTag cells, indicating TGR5 is mainly target for them. These results indicated that TGR5 might be the main target responsible for activity of Hesperetin and Naringenin.

### 3.5 Hesperetin and Naringenin promoted GLP-1 secretion through activation of TGR5 receptor in L-cells

**FIGURE 5:**
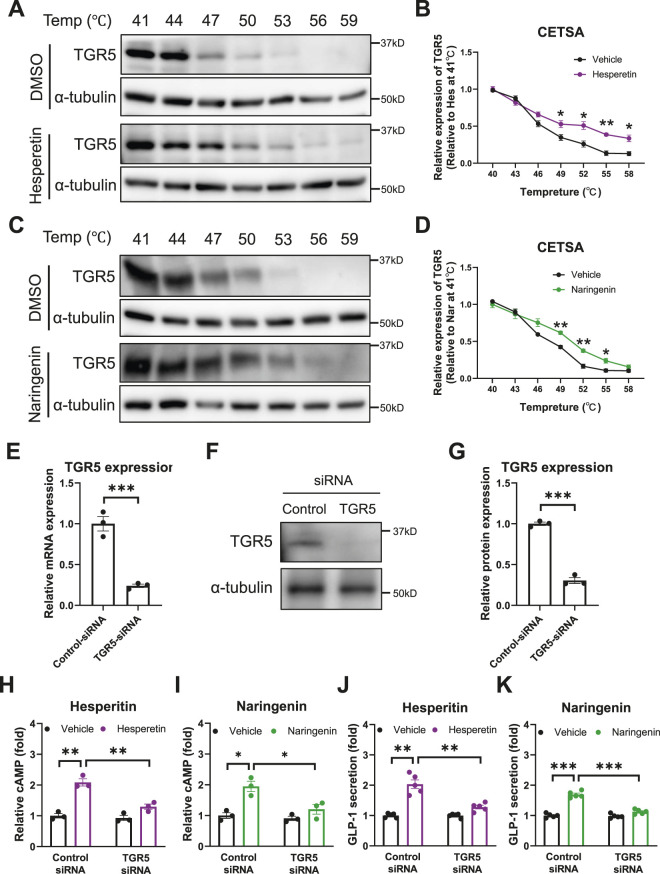
Hesperetin and Naringenin promoted GLP-1 secretion through activation of the TGR5 receptor. **(A–D)** Western blot images and quantitative analysis of TGR5 protein levels at temperatures ranging from 41 °C to 59 °C under a short exposure of Hesperetin **(A,B)** or Naringenin **(C,D)** in CETSA assay (n = 3). **(E–G)** GLUTag cells were transfected with control or TGR5 siRNA for 48 h, and the knockdown efficiency was evaluated by RT-qPCR **(E)** and Western blot **(F,G)** analysis (n = 3). **(H,I)** Intracellular cAMP levels from GLUTag cells in response to 10 μM Hesperetin **(H)** or 10 μM Naringenin **(I)** (n = 3). **(J,K)** GLP-1 secretion from GLUTag cells transfected for 48 h with control or mouse TGR5 siRNA in response to 10 μM Hesperetin **(J)** or 10 μM Naringenin **(K)** (n = 5). *p < 0.05, **p < 0.01, ***p < 0.001 by Mann–Whitney U‐test. Data are expressed as the mean ± SEM.

To validate TGR5 as a direct molecular target for Hesperetin and Naringenin, we performed cellular thermal shift assay (CETSA). The CETSA results demonstrated that GCK levels declined at 47 °C and were almost undetectable at 56 °C in vehicle-treated cells, whereas both Hesperetin and Naringenin significantly reduced degradation (Figures 5A–D). These data indicated that Hesperetin and Naringenin could bind with TGR5. To confirm our speculation that Hesperetin and Naringenin promoted GLP-1 secretion through activation of TGR5 receptor in GLUTag cells, we next measured physiological effect of Hesperetin and Naringenin on TGR5 activity in TGR5-siRNA treated GLUTag cells. The qPCR and Western blot results demonstrated the successful downregulation of mRNA and protein levels of TGR5 with administration of siRNA-TGR5 compared with those of siRNA-control treated group in GLUTag cells (Figures 5E–G). Since TGR5 is a G protein-coupled receptor that primarily couples with Gs proteins leading to increased intracellular cAMP levels (Lun et al., 2024), we first detected level of Gs-coupled cAMP. Hesperetin and Naringenin significantly enhanced cAMP level in control-siRNA treated GLUTag cells, however, neither of them changed cAMP level in TGR5-deficient GLUTag cells (Figures 5H,I). Then we analyzed GLP-1 secretion from TGR5 deficient GLUTag cells in response to Hesperetin or Naringenin. The GLP-1 release was enhanced by Hesperetin or Naringenin from the control-siRNA treated GLUTag cells, however, such increment was significantly decreased upon reduction of TGR5 expression by TGR5 siRNA (Figures 5J,K). These results suggested that Hesperetin and Naringenin promoted GLP-1 secretion through activating TGR5 receptor in murine L cells.

### 3.6 Short-term administration of Hesperetin and Naringenin promoted GLP-1 secretion and alleviated hyperglycemia in HFD-induced T2D mice

**FIGURE 6:**
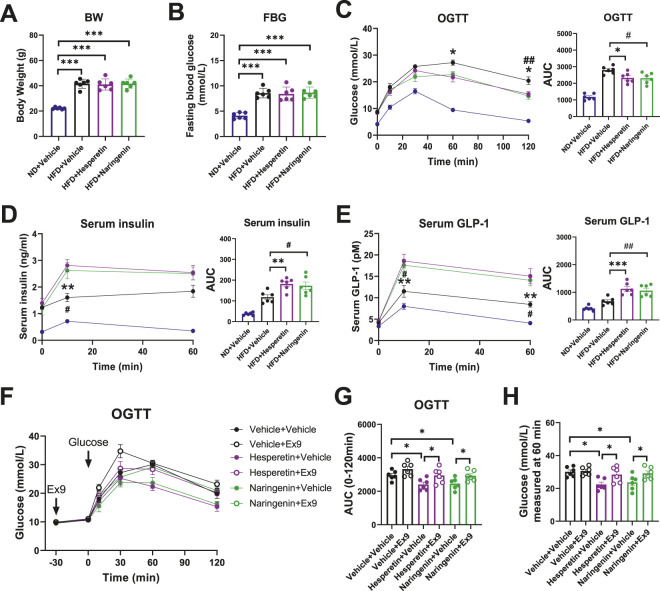
Effect of short-term administration of Hesperetin and Naringenin on HFD-induced obese diabetic mice. **(A,B)** Body weight **(A)** and fasting blood glucose level **(B)** of ND mice and HFD-induced obese diabetic mice before treatment. n = 6 for each group. ***p < 0.001 by one-way ANOVA with Turkey’s test. **(C,F)** Phenotype of ND mice and HFD-induced obese diabetic mice receiving a single dose of 100 mg/kg Hesperetin, 100 mg/kg Naringenin or vehicle. OGTT performed after an oral glucose gavage (2 g/kg): **(C)** Blood glucose (left) and corresponding AUC (right); **(D)** Serum insulin levels (left) and the AUC (right); **(E)** Serum GLP-1 levels (left) and the AUC (right). n = 6 for each group. *p < 0.05, **p < 0.01, ***p < 0.001 compared between vehicle and Hesperetin, ^#^ *p* < 0.05, ^##^ *p* < 0.01 compared between vehicle and Naringenin, by one-way ANOVA with Turkey’s test. **(F–H)** OGTT (2 g/kg) in mice pretreated with GLP-1R antagonist Exendin-(9-39) (Ex9, 50 μg/mouse): **(F)** Blood glucose in whole period; **(G)** Area under the curve (AUC) corresponding to OGTT; **(G)** Blood glucose levels at 60 min time point in OGTT. n = 6 for each group. *p < 0.05 by Mann–Whitney U‐test. Data are presented as mean ± SEM.

To further examine the role of Hesperetin and Naringenin in GLP-1 secretion *in vivo*, we detected serum GLP-1 level by using HFD induced diabetic mice. Compared with ND fed mice, HFD induced diabetic mice showed markedly increased body weight and fasting blood glucose indicating successful T2D mouse model (Figures 6A,B). We detected acute effect of Hesperetin and Naringenin on blood glucose regulation using HFD-induced T2D mice with comparable body weight and fasting blood glucose levels, as well as ND mice as a baseline reference (Figures 6A,B). Impaired glucose tolerance and serum insulin level were significantly improved after administration of Hesperetin or Naringenin (Figures 6C,D). In addition, serum GLP-1 level in response to glucose was notably increased in the presence of Hesperetin or Naringenin, and Hesperetin appeared to have a comparatively greater effect on stimulating GLP-1 secretion than Naringenin (Figure 6E). To further support our findings, we tested whether Exendin-(9-39) (Ex9), a GLP-1 receptor antagonist, could abolish the improvement of glucose metabolism in Hesperetin or Naringenin treated mice. In line with our expectation, compared with improved effect on hyperglycemia in Hesperetin or Naringenin treated mice, Ex9 significantly suppressed the improvement in hyperglycemia observed in Hesperetin- or Naringenin-treated mice, leading to elevated blood glucose levels (Figures 6F,G). Notably, 60 min after glucose dosing, Hesperetin/Naringenin + Ex9 group had significantly higher blood glucose than Hesperetin/Naringenin + vehicle group respectively (Figure 6H). These findings suggested that short-term administration of Hesperetin and Naringenin could improve impaired glucose tolerance through enhancing GLP-1 secretion in response to glucose ingestion *in vivo*.

### 3.7 Long-term administration of Hesperetin and Naringenin improved HFD induced obesity and diabetes

**FIGURE 7:**
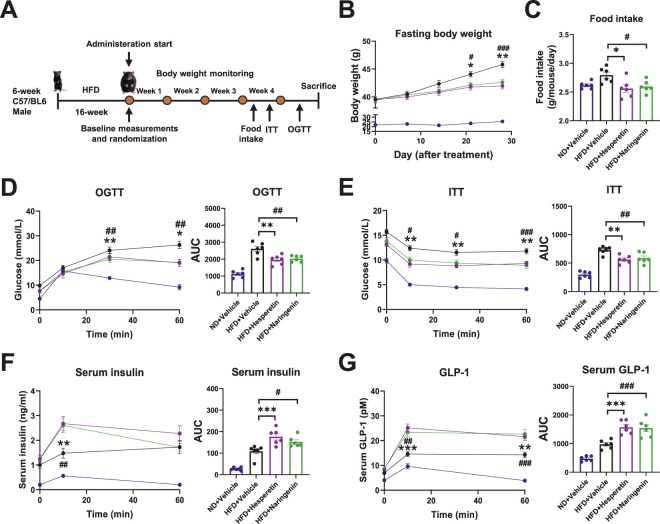
Effect of long-term administration of Hesperetin and Naringenin on HFD-induced obese diabetic mice **(A)** Flowchart of long-term study, the procedure was introduced in method. **(B–G)** Phenotype of ND mice and HFD-induced obese diabetic mice treated with 100 mg/kg Hesperetin, 100 mg/kg Naringenin or vehicle. **(B)** Body weight change during the 4-week treatment period. **(C)** Food intake in the last week. **(D)** OGTT with 2 g/kg glucose (left) and corresponding AUC (right). **(E)** ITT with 0.75U/kg insulin (left) and corresponding AUC (right). **(F)** Serum insulin levels (left) and the AUC (right). **(G)** Serum GLP-1 levels (left) and the AUC (right). *p < 0.05, **p < 0.01, ***p < 0.001 compared between vehicle and Hesperetin, ^#^ *p* < 0.05, ^##^ *p* < 0.01, ^###^ *p* < 0.001 compared between vehicle and Naringenin, by one-way ANOVA with Turkey’s test, ns means not significant. Data are presented as mean ± SEM.

To investigate the therapeutic effects of Hesperetin and Naringenin on diabetes and obesity, we examined the influence of 28-day once daily treatment with Hesperetin or Naringenin on HFD-induced type 2 diabetic mice (Figure 7A). The initial fasting body weight of HFD-fed mice was similar for every group which is obviously higher than ND-fed mice. However, following 4-week daily oral administration, both Hesperetin- and Naringenin-treated HFD mice were significantly attenuated body weight gain compared to the vehicle group (Figure 7B), accompanied with a markable reduction in cumulative food intake (Figure 7C). To further assess their impact on glycemic control, we performed OGTT and ITT. Both Hesperetin- and Naringenin-treated mice exhibited significantly improved impaired glucose tolerance and insulin sensitivity, as evidenced by lower blood glucose before and during OGTT (Figure 7D) and a greater glucose-lowering response to exogenous insulin in ITT (Figure 7E). Consistent with our acute secretion findings, chronic administration led to elevated levels of serum insulin (Figure 7F) and GLP-1 (Figure 7G) in response to glucose. These results indicated that long-term administration of Hesperetin and Naringenin improved HFD induced obesity and diabetes.

## 4 Discussion

The global health burden of T2D underscores urgent need for innovative therapeutic strategies that are both effective and accessible (Weinberg Sibony et al., 2023). While synthetic GLP-1 analogs and DPP-4 inhibitors are powerful treatments, concerning about cost, injectable administration, and gastrointestinal side effects limit their use (Fu et al., 2024). Enhancing the ability to secrete GLP-1 represents a complementary and potentially more sustainable strategy (Watkins et al., 2021). In this study, we directly identified the citrus flavonoids Hesperetin and Naringenin as novel GLP-1 secretagogues by using a reporter cell line. Further study clarified that they enhanced GLP-1 secretion through activation of TGR5, which in turn elevated cAMP-PKA signaling pathway and calcium influx, thereby improving obesity and glucose metabolism under obese diabetic conditions. By offering a natural means to augment the endogenous incretin system, these flavonoids present a compelling new avenue for the dietary management of type 2 diabetes and obesity.

GLP-1 controls meal-related glycemic excursions through augmentation of insulin and inhibition of glucagon secretion, which inhibits gastric emptying and food intake (Drucker, 2018). Hesperetin/Naringenin increased GLP-1 secretion from gut enteroendocrine cells in response to glucose stimulation. The activation of pathways such as increased cAMP-PKA signaling pathway and amplified glucose-triggered calcium influx provides a comprehensive mechanism (Watkins et al., 2021; Sun et al., 2017). The abolition of these effects by specific inhibitors such as H89 for PKA and nifedipine for Ca^2+^, underscores the interdependence of these signaling cascades in mediating the robust secretion of GLP-1.

Increased cAMP level and activated calcium ion channels were similar consequences of GPCRs activation (Thomas et al., 2009; Cho et al., 2025). To find out the exact target and rigorously exclude potential off-target effects, we systematically evaluated agonist activity against a panel of related receptors. Hesperetin nor Naringenin significantly activated TGR5 by dual-luciferase reporter assay, however, neither Hesperetin nor Naringenin activated reporters for other key L-cell-relevant GPCRs (GPR40, GPR119, GPR120) or the nuclear bile acid receptor FXR. These findings confirmed TGR5 as a molecular target, aligning with prior reports linking TGR5 to cAMP/PKA-driven GLP-1 exocytosis (Liu et al., 2024; Yang et al., 2023). This conclusion was further corroborated by loss-of-function experiments, where TGR5 knockdown abolished the stimulatory effects of both flavonoids on cAMP production and GLP-1 secretion.

The efficacy of Hesperetin and Naringenin translated impressively from *in vitro* models to an *in vivo* setting. In diet-induced obese diabetic mice, a single dose of Hesperetin or Naringenin could improve glucose tolerance and elevate serum GLP-1 levels after an oral glucose challenge while such improved effect on glucose metabolism was demonstrated significant biological activity. Importantly, chronic administration over 4 weeks translated this acute secretory boost into sustained metabolic benefits, including attenuated weight gain and food intake, improved glucose tolerance, and enhanced insulin sensitivity, which were consistent with the effects of elevated GLP-1 (Son et al., 2025). The sustained elevation of glucose-stimulated GLP-1, following chronic treatment reinforces the selective action on TGR5-expressing L-cells rather than a generalized enteroendocrine effect. This hormone-specific profile, combined with the metabolic improvements, highlights the therapeutic potential of these flavonoids as nutraceutical agents.

In this study, we did not perform a comprehensive pharmacokinetic analysis since the absorption and disposition of Hesperetin and Naringenin have been extensively characterized elsewhere (Kanaze et al., 2007). Instead, we based our selection of drug concentrations on published pharmacokinetic data: oral administration of 50–150 mg/kg of either compound in humans or rodents yields peak plasma levels of approximately 1–10 μM, which fall well within the range shown to activate TGR5 in our *in vitro* assays (Najmanova et al., 2020). Thus, the effective concentrations used in our study are physiologically achievable in the gut lumen, the primary site of L-cell exposure.

Overall, our findings not only provide a mechanistic basis for the purported antidiabetic benefits of citrus bioactive but also highlight these flavonoids as promising candidates for the development of nutraceutical or therapeutic agents aimed at enhancing endogenous GLP-1 secretion.

## Data Availability

The original contributions presented in the study are included in the article/supplementary Material, further inquiries can be directed to the corresponding authors.
